# Prenatal Diagnosis of β-Thalassemias and Hemoglobinopathies.

**DOI:** 10.4084/MJHID.2009.011

**Published:** 2009-11-15

**Authors:** Maria Cristina Rosatelli, Luisella Saba

**Affiliations:** Dipartimento di Scienze Biomediche e Biotecnologie Università degli Studi di Cagliari. Italy

## Abstract

Prenatal diagnosis of β-thalassemia was accomplished for the first time in the 1970s by globin chain synthesis analysis on fetal blood obtained by placental aspiration at 18–22 weeks gestation. Since then, the molecular definition of the β-globin gene pathology, the development of procedures of DNA analysis, and the introduction of chorionic villous sampling have dramatically improved prenatal diagnosis of this disease and of related disorders. Much information is now available about the molecular mechanisms of the diseases and the molecular testing is widespread. As prenatal diagnosis has to provide an accurate, safe and early result, an efficient screening of the population and a rapid molecular characterization of the couple at risk, are necessary prerequisites. In the last decades earlier and less invasive approaches for prenatal diagnosis were developed. A overview of the most promising procedure will be done. Moreover, in order to reduce the choice of interrupting the pregnancy in case of affected fetus, Preimplantation or Preconceptional Genetic Diagnosis (PGD) has been setting up for several diseases including thalassemias

## Introduction:

β-thalassemias and hemoglobinopaties are among the commonest autosomal recessive diseases with a high frequency in population of the Mediterranean area, the Middle East, the Indian subcontinent, the Far East, Tropical Africa and the Caribbean.^1^ However, in the last decades, the steady migratory flows have rendered these pathologies much more widespread, thus representing a general public health problem. In the ‘70s the set-up of globin chain synthesis analysis for the detection of little amount of β-chains in fetal blood during the 18th-22th week gestation^2^ has allowed the development of screening programs of the general population, based on the identification of the couple at risk, and, in addition, the offer of prenatal diagnosis testing. At that time the thalassemic patients had limited lifespan and prenatal diagnosis represented the only option for the control of the disease. Such programs first started in Sardinia, Continental Italy, Cyprus e Greece.^3^,^4^,^5^,^6^

Prenatal diagnosis on fetal blood, even if represented for couple at-risk an opportunity to generate healthy sons, was not easily accepted. The late gestational age in which fetal diagnosis was carried out, the risk of misdiagnosis due to a not clear cut-off between some heterozygotes and affected fetuses, the high risk of miscarriage due to the sampling procedures, made indeed the procedure difficult to accept from the couples.

The continuous advances in the knowledge of the molecular pathology of the disease, the discovery of restriction fragment length polymorphisms (RFLP) linked to the β-like globin gene, the development of methodologies for mutation detection and the application of the villocentesis for the recovery of nucleated fetal cells, allowed a fast improvement both in feasibility and acceptability of prenatal diagnosis. For a short period, in the eighties, the diagnosis of thalassaemia was obtained either indirectly by linkage analysis using RFLP at the β-globin gene cluster^7^ or directly by oligonucleotide hybridisation on electrophoretically separated DNA fragments^8^ or by enzymatic digestion of mutated sites. A major impulse has been given by the PCR technology that allowed the development of a number of procedures, for easier mutation detection, as well as the development of both PGD and non invasive prenatal diagnosis procedures. Nowadays thalassaemias are detected directly by the analysis of amplified DNA from fetal trophoblast or, more rarely, from amniotic fluid cells.

In this review we will delineate current procedures for prenatal and preimplantation diagnosis of thalassemias as well as the most promising approaches for non-invasive prenatal diagnosis.

## Prenatal Diagnosis

### Detection Methods:

Definition of molecular defect in both parents is a prerequisite for prenatal diagnosis of the disease.

The majority of defects affecting the β-globin gene are point mutations that occur in critical areas for its function, or single/few base addition/deletion that change the frame in which triplets are translated into protein. Very rarely β-thalassemia results from gross rearrangement in the β-globin gene cluster. In spite of the marked molecular heterogeneity, a limited number of molecular defects are prevalent in every at risk population. This may be very useful in practice, because a panel of most frequent mutations to be searched for can be designed according to the carrier’s ethnic origin. 9

Known mutation detection is carried out by a number of PCR-based techniques. Among them, the most commonly used are the primer-specific amplification (ARMS, Amplification Refractory Mutation System) and the reverse oligonucleotide hybridisation with specific oligonucleotide probes (RDB, Reverese Oligonucleotide-probe analysis).

### Primer-specific Amplification:

The method is based on the principle that a primer carrying a mismatch in its 3’ region cannot anneal on its template. With this method, the target DNA fragment is amplified in two separate PCR reactions using a common primer and either of the two following primers: one complementary to the mutation to be detected (β-thalassemia primer) and one complementary, at the same position, to the normal DNA (normal primer). Normal DNA is amplified only by the normal primer while DNA from homozygotes only by the β-thalassemia primer and DNA from heterozygotes by both primers. A different sized fragment of the β-globin gene is simultaneously co-amplified as an internal control of the PCR reaction.^10^ The method is very simple as it requires, for each mutation to be searched, only two PCR reactions followed by agarose gel electrophoresis. A further improvement of the methodology can be obtained by multiplexing the primers for more than one mutation. In good hands the method is very safe and particularly useful in fetal DNA analysis to search for mutations previously detected in the parents.

### Reverse Oligonucleotide Hybridization:

When the spectrum of mutations to be searched is complex, ARMS is not the most appropriate method. In this case RDB results to be more informative and efficient. The method uses membrane-bound allele-specific oligonucleotide probes that hybridize to the complementary sequence of the PCR product prepared using patient DNA as starting template.^11^ In this format, multiple pairs of normal and mutant allele-specific oligonucleotides can be placed on a small strip of membrane. Hybridization with PCR-amplified β-globin gene is able to detect, in a single procedure, any of the mutations screened. Up to 20–30 mutations have indeed been screened in one single step and several commercial kits are available to detect the most common beta thalassemia mutations in Mediterranean population.

### Other Known Mutation-detection Procedures:

Several other methods have been developed to search for known mutations, i.e. oligonucleotide ligation assay,^12^ restriction enzyme digestion of PCR products,^13^ however some of them have been abandoned in routine diagnostics as they are less informative or more complex.

In recent years a real time PCR assay has been successfully applied to both carriers screening and prenatal diagnosis.^14^ This is a one-step method that is based on the use of fluorescent hybridization probes followed by a melting curve analysis. The method, which allows the simultaneous multiple mutation detection, has been successfully applied also to the detection of maternal contamination. In spite of these advantages its use is still limited as it needs a dedicated apparatus as well as an accurate population-based design of detection probes.

Technically, we can realistically predict further simplification and fully automation of the procedures for the detection of the β-thalassemia mutation in carrier screening and prenatal diagnosis. Nowadays a number of systems for molecular detection of the β-thalassemia mutations is commercially available, which are not completely automated and quite expensive. Among them, the oligonucleotide microchip based-assays have been proposed many times for the large-scale detection of mutations in genetic diseases, including β-thalassaemia.^15^ Given the alternative features of high throughput and automation, the DNA chip has the potential to become a valuable method in future applications of mutation detection in medicine. At the moment the technology, developed several years ago, is not jet transferred in the clinical practice, due to the higher costs and to the lower analytical sensitivity and specificity.

### Unknown Mutations Detection:

When carriers escape to the above mutation detection approaches, further investigations need to be carried out by alternative methods which uncover the presence of unknown mutations by scanning the whole gene. Denaturing gradient gel electrophoresis (DGGE),^16^,^17^,^18^ denaturing High Pressure liquid Chromatography (dHPLC) and Single Strand Conformation Polimorphism (SSCP)^19^ are the most widely used in the last years, followed by direct sequencing analysis^20^ which characterizes the undefined mutation found by these methods. Nowadays, considering the small size of the β-globin gene (1,8kb), the simplified technologies available and the reduced costs of analysis, direct sequencing, based on cycle sequencing with fluorescent dye terminators and automated capillary DNA sequencing technology, seems to be the faster and most useful approach to detect unknown thalassemia mutations.

If a mutation is not detected by sequence analysis, we search for the presence of small deletions by polyacrylamide gel electrophoresis of amplicons designed for the most frequent small deletional defects of the β-globin gene (gap PCR). Furthermore, the presence of larger deletions of the cluster may be identified by Southern-blotting or more recently by Multiple Ligation-dependent Probe Amplification (MLPA) for which a commercial kit is available (SALSA MLPA KIT P102 HBB-MRC Holland).

In a very limited number of cases, direct sequencing from position -600 to 60 bp downstream from the β-globin gene and methods for deletion detection, failed to detect the disease causing defect. In these cases, the molecular defect may reside either in the locus control region of the β-globin gene cluster, or in one of the genes, outside the β-globin gene region, encoding for regulatory proteins acting in trans on the function of the β-globin gene. Very recently it has been proved that the β-thalassaemia like phenotype could be caused by the coinheritance of a β-globin gene defect and a duplication of the α- globin gene cluster, which results in an excess of α chain. In these selected cases, the characterization of these α-globin gene rearrangements (SALSA MLPA KIT P140-B2 HBA-MRC Holland) can be routinely carried out with success by MLPA analysis.

### Genetic Counseling of the Couple at Risk:

Both members of the couple at risk are counseled in a non directive way. The nature of the disease, the implications of being carriers and the reproductive choices are analyzed, specifically those concerning birth control, including prenatal or preimplantation diagnosis and the possibility, in case of affected fetus HLA compatible, to not interrupt pregnancy As for fetal testing, detailed information is offered regarding the risk of fetal mortality, the risk of misdiagnosis, and the mortality and morbidity of an abortion in case of affected fetus.

### Fetal DNA Sampling:

Fetal DNA for analysis can be obtained from either amniocytes or chorionic villi. At present the most widely used procedure is chorionic villi sampling, because of the clear advantage of being carried out during the first trimester of pregnancy, generally at the 10th–12th week of gestation.^21^, ^22^, ^23^ The risk of fetal mortality associated with both methods is in the order of 1–2%. Chorionic villi may be obtained transcervically or transabdominally, the last being most widely used, mainly because it has a low infection rate and a lower incidence of amniotic fluid leakage. Moreover it is a simple procedure, largely preferred by pregnant women, that can be carried out also in late gestational age.

Samples obtained by villocentesis need to be accurately dissected under inverted microscope in order to remove maternal decidua, that represent the major cause of diagnostic error in prenatal diagnosis of monogenic diseases,

### Fetal DNA Analysis:

Fetal DNA is analysed using the same methods described above for the detection of known mutations during carrier molecular screening. To limit the possibility of misdiagnosis, we analyse chorionic villous DNA with two different procedures: i.e. RDB hybridisation and primer-specific amplification, using distinct couples of primers.

Misdiagnosis may occur for several reasons: failure to amplify one copy of the target DNA fragment, mispaternity, maternal contamination, and sample exchange. Misdiagnosis for failure of DNA amplification is obviously limited by the double approach described above. To avoid misdiagnosis due to maternal contamination as well as mispaternity and/or sample exchange, a fetal DNA microsatellite analysis is usually performed to verify the presence of one allele from each parent.^9^ In our hands, by the above mentioned PCR-based procedures, no misdiagnoses have occurred in more than 5000 cases.

Currently, prenatal diagnosis is a widely applied and well accepted procedure. Among the patients screened we have found an acceptability of 99,3% for early prenatal diagnosis by CVS. This data, if compared with previously utilized procedures such as fetal blood sampling, with an acceptability of 93,2%, and 96,4% by amniocentesis, demonstrates how the acceptance of the procedure depends on its precocity.^22^

**Figure 1.**
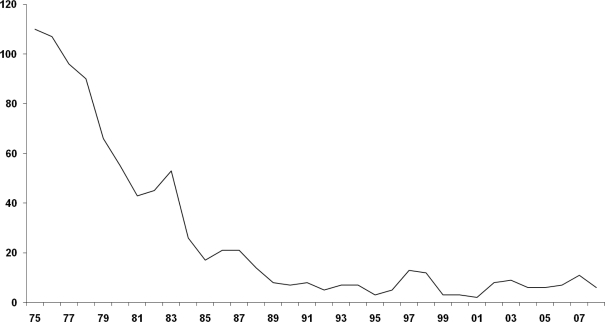
Fall in the birth rate of homozygous β-thalassemia in Sardinia.

The screening program in the Mediterranean countries have proven to be very successful in reducing the number of thalassemia patients. In Sardinia, thalassemia major was present in 1 in 250 births, and has declined to 1 in 4000 births (Figure1). Other countries in which such thalassemia programs have been introduced also show similar trends.

### Preimplantation and Preconceptional Genetic Diagnosis:

The progress in assisted reproduction and molecular genetics techniques, particularly the advent of PCR that has made possible to analyze the genotype of a single cell, has paved the way for preimplantation genetic diagnosis (PGD).^24^,^25^

This technique was introduced as an option for avoiding the decision to terminate an established pregnancy diagnosed as affected by conventional approaches.

The term preimplantation genetic diagnosis describes those procedures which involve the removal of one or more nuclei from ovocytes (polar bodies) or embryos (blastomeres of trophectoderm cells) to test for mutation in the target gene or aneuploidy before transfer.

PGD requires that couples at risk undergo in vitro fertilization (IVF) even if not infertile and for this reason a multidisciplinary approach including an appropriate genetic counseling and the referral to both a fertility clinic and to a high specialized molecular genetics laboratory is mandatory.

Counseling for couples considering PGD must include additional information regarding at least the risk associated with IVF procedures and with embryo biopsy, the technical limitations of DNA analysis, including the risk of failure of the procedure as well as that of misdiagnosis, and the need of subsequent prenatal diagnosis to confirm the result. Beyond that, the possibility that no embryos may be transferred and the dispositions of embryos not transferred have also to be seriously considered.

### Cell Biopsy:

Preimplantation may be carried out by either cleavage-stage biopsy of 1–2 blastomeres, from an eight-cell embryo three days after in vitro fertilization carried out by ICSI (Intracytoplasmatic Sperm Injection), or by the biopsy of polar bodies.

For cleavage-stage biopsy the embryo is grown in vitro until it reaches a six-eight cell stage, which usually occurs on the third day after insemination. Polar bodies diagnosis, pioneered by Verlinsky and his group in ’90,^26^ is based on the analysis of the first polar body of unfertilized eggs,^27^ and may lead to distinguish between unfertilised eggs that carry the defective gene and those without the defect. The successive sampling and analysis of the second polar body that is extruded from the oocyte after fertilization and completion of the second meiotic division, is carried out in order to avoid misdiagnosis due to the high rate of recombination that happens during the first meiosis. By fertilizing in vitro only the eggs without the defect and replacing them in the mother, a successful pregnancy with a normal fetus can be obtained. Recently a preconceptional genetic diagnosis based on the analysis of only the first polar body has been proposed for countries in which the use of PGD and manipulation of embryos is prohibited.^28^ This approach although permitting to avoid the manipulation, cryopreservation and/or discard of sovranumerary and/or affected embryos, shows several problems: the need to obtain more then 10–12 oocytes, the increased risk of diagnostic error and the increase of the technical difficulties.

Blastocyst biopsy, even if it has the advantage to provide a higher number of cells,, is at present more rarely used because of the difficulties of the embryos to reach this stage in IVF programs.

The cleavage-stage biopsy of blastomeres from a eight-cell embryo is the most frequently used PGD procedure all over the world.

### Detection Methods:

Methods for mutation detection in PGD are always based on multiple steps of PCR. Mutations are detected in PCR products by various methods that combine speed, analytical sensitivity and specificity. In particular, a first round of multiplex PCR is performed to amplify both the β-globin gene region including the mutation and one or more polymorphic loci. Secondly, two separated nested PCR reactions are performed to amplify the two or more selected genomic regions. Finally, the polymorphic alleles are directly detected by capillary electrophoresis of the amplified fragment, while the presence of β-globin gene mutations are identified by a subsequent minisequencing reaction.^29^ This approach is expressely designed to detect the presence of the β-globin gene mutations and to monitor, in the same sample, the presence of contamination as well as the eventual allele dropout that represent the most frequent causes of error in PGD.

### Quality Control:

For both techniques a prenatal diagnosis by villocentesis is recommended in order to avoid diagnostic errors.

Successful pregnancies following the transfer of human embryos in which the β-globin gene defect has been excluded, occur only in 20–25% of cases and the birth rate of a child is even lower. Due to the low birth rate most women have to undergo PGD several times in order to give birth to a healthy child.^30^

Transfer of no more than 1–2 embryos is strongly recommended in order to avoid multiple pregnancies.^31^ Elective Single Embryo Transfer (eSET) is in fact a well established procedure which has demonstrated to ensure a better prognosis of IVF patients.^32^

### PD or PGD?

Among clinical geneticists there have been much discussion about the main goal of PD. Some have argued that the main aim is to avoid the birth of an affected child. Others have emphasized the reproductive confidence and the purpose of informing couple at risk about the status of the fetus. Several studies indicate that if there is no PD option, a large proportion (up to 50%) of the couples at high risk of an affected child refrain from pregnancy despite their wish to reproduce. When PD is possible many more at-risk couples dare to embark on a pregnancy.

Most experts consider PGD as an additional option for couples at risk and not as a replacement for conventional prenatal diagnosis. PGD is still considered a highly specialized experimental procedure with limited results, mainly dedicated to couples against abortion for ethical and religious reasons and to a small proportion of couples, who have experienced repeated abortion, that ask for referral for this procedure.

At present its use in routine monitoring of pregnancies at risk is precluded by the technical demand for these procedures, the difficulty in organizing the service, and the high costs.

Simplification of preimplantation and preconception genetic diagnosis, together with an increase in the pregnancy rate, may lead to a more extensive use of the procedure in the future.

## Non Invasive Prenatal Diagnosis (NIPD):

### Analysis of Fetal Cells in Maternal Blood

In the sections below the most significant studies, which have been carried out in this field of research, are briefly summarized. The most relevant results have been grouped in three different sections, according to the different cell type in which they have been acquired. A separate section is dedicated to the the NIPD of β-thalassemia.

### Trophoblasts:

The first evidence that fetal cells circulate in maternal peripheral blood dates back to 1893 when George Schmorl observed the presence of placentally derived trophoblasts in the lungs of 17 autopsied women affected by severe eclampsia.^33^

In 1959 Douglas^34^ established that migration of trophoblasts is a normal process during pregnancy and twenty five years later, Covone et al^35^ demonstrated that these cells could be detected in healthy pregnant women as early as six weeks gestation. They also found that an increased concentration of trophoblast cells were frequently present in women affected by preeclampsia. Further studies have established that trophoblasts are entrapped in the maternal lungs and rapidly removed from the pulmonary circulation.^36^

Trophoblast-specific cell-surface antigens have not yet been characterized and several experimental evidences have shown that the H315, initially described as the specific antigen for trophoblasts, is indeed adsorbed in maternal leucocytes.^37^

These are some of the reasons why, in recent years, trophoblasts are no longer considered as the best target cells for non invasive prenatal diagnosis. Nevertheless, this line of research has not yet completely abandoned as the characterization of trophoblast-specific antigens is one of the objectives of the SAFE (Special Non-Invasive Advances in Fetal and Neonatal Evaluation) Network (for more information please visit www.safenoe.org).

### Lymphocyte:

Fetal lymphocytes are the second cell type which have been extensively studied as possible source of fetal DNA. In 1969 Walknowska et al^38^ detected for the first time 46,XY karyotype cells in maternal peripheral blood of women bearing male fetuses. Ten years later Herzenberg and collegues described the use of FACS (Fluorescent Activated Cell Sorting) as a method for the enrichment of fetal lymphocyte expressing the HLA-A2 paternal antigen.^39^ Detection of Y chromosome was then obtained in the enriched cells deposited directly onto microscope slides, thus confirming their fetal origin.

Unfortunately other groups have failed to replicate these results with success, even if cytogenetic analysis were carried out in fetal cells flow sorted on the basis of several HLA differences and by using monoclonal antibodies.

In the same years further studies demonstrated that lymphocytes were not removed from maternal circulation after delivery. One of the earliest study who provided the first evidences that fetal lymphocytes persist in maternal circulation one year after delivery was published in 1974.^40^ Several years later Bianchi et al described the presence of fetal progenitor cells 27 years after delivery.^41^

For these reasons also lymphocytes, as trophoblasts, became an unattractive candidate for non invasive prenatal diagnosis.

### Erythroblasts:

One of the main advantage to study fetal erythroid cells is that they are nucleated, terminally differentiated short-lived cells and for this reason they do not persist in maternal circulation for long time after delivery. Furthermore, first primitive erythroblasts appear in embryonic bloodstream around the four-five weeks gestations so they can be detected early during gestation.

Nevertheless, their isolation from maternal peripheral blood is still problematic because of their rarity and the lack of a fetal specific antibody.

In 1990 Bianchi^42^ firstly described a method for fetal nucleated erythroid cells enrichment, which was based on the combined use of flow sorting (FACS) and CD71 transferrin receptor, highly expressed in erythroid cells. Two years later Gänshirt-Ahlert et al^43^ obtained similar results by using a newly detection system called MACS (Magnetic Cell Sorting) which is based on the use of antibodies labeled with magnetic beads.

**Figure 2.**
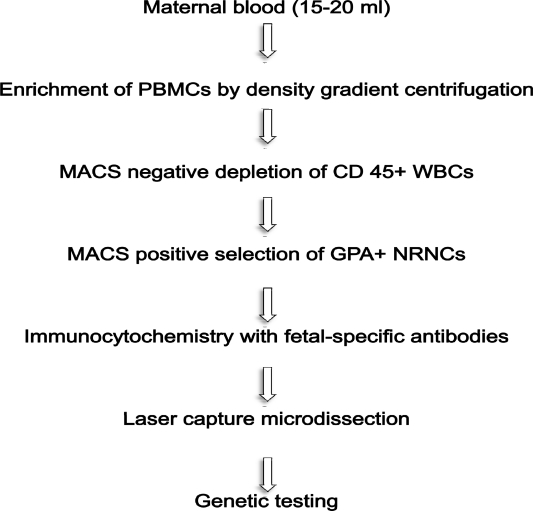
In the present workflow is resumed one of the commonly used strategies for isolating fetal NRBCs from maternal peripheral blood. **PBMC** *Peripheral Blood Mononuclear Cell*, **WBC** *White Blood Cell*, **NRBC** *Nucleated Red Blood Cell*, **MACS** *Magnetic Activated Cell Sorting*.

**Figure 3.**
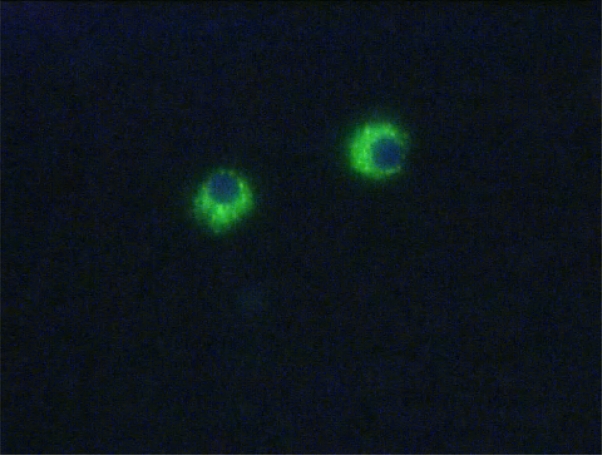
Erythroblasts enriched from maternal blood and stained with monoclonal antibody for ɛ-globin coniugated with FITC. Nuclei are counterstained with DAPI.

Since then, both system have been extensively improved and used, by several groups, following different approaches which can consist in the positive selection of CD71 and/or glycophorin-A fetal cells and/or in the negative depletion of CD45 maternal cells. Usually, in both cases, a previous density (Ficoll or Histopaque) gradient centrifugation step is carried out to remove non nucleated maternal erythrocytes. A schematic workflow resuming one of the strategies used for isolating fetal NRBCs from maternal peripheral blood is represented in figure 2. Finally both MACS and FACS sorted cells are labeled with fluorescent antibodies which recognize embryonic (ɛ, ζ) or fetal (γ) hemoglobin chains and are eventually subjected to FISH analysis for chromosome Y detection. An example of positive labeling with the antibody for ɛ-globin coniugated with FITC is shown in figure 3. Molecular characterization can be eventually carried out in positive fluorescent cells isolated by laser microdissection.

Even with the high progress made in last twenty years in this field, the methods for erythroblasts enrichment are still limited as they mostly result in the recovery of fetal samples with low yield (FACS) and scarce purity (MACS), being variably contaminated by maternal cells.

For these reasons in recent years several studies have been addressed to the proteomic field with the attempt to characterize novel fetal erythroblast cell-specific surface markers.

For example, bi-dimensional electro-phoresis coupled with mass spectrometry has allowed the identification of 22 proteins, differentially expressed in sickle erythrocytes in comparison to healthy erythrocytes, and the detection of proteins up- or downregulated in fetal erythroid cells in comparison to their adult counterparts. Some of these results have been published as a full-patent application and the data concerning the new antibodies developed against these new targets expect to be validated in large samples of maternal blood.^44^

In addition, further developments in fetal cells recovery are expected to be obtained through the application of micro-fluidic rare-cell capture technologies 45 which are being developed to detect not only fetal but also cancer as well as other rare cells in biologic fluids.

### Analysis of fetal cells in maternal blood and Non Invasive Prenatal Diagnosis (NIPD) of β Thalassemia:

Despite the difficulties encountered to find the best target cell and the best method for their enrichment and isolation, several attempts have been made in last twenty years, to transfer the results of these researches into clinical practice. Unfortunately the lack of reproducibility of experiments hardly makes the isolation of fetal cells from maternal blood as a first choice method for NIPD of monogenic disorders. Below the most significative results obtained in NIPD of β-thalassemia are briefly summarized.

The first example of non invasive prenatal diagnosis of haemoglobinopathies was described in 1990 by Camaschella et al.^46^ The genetic test was carried out in three selected couples where the mother was carrier of β-thalassemia and the father of the Hb Lepore-Boston trait. The absence/presence of the paternal trait was successfully detected in PCR amplified samples (DNA extracted from T-lymphocyte depleted cells) and autoradiography. In this case the “enriched fetal cell” samples were obtained by incubating Ficoll-separated cells of the mother with the CD 3-specific MoAb Leu 4 and then separating the positive cells with goat-anti-mouse immunoglobulin G (1gG)-coated immunomagnetic beads.

In those years most of the studies were addressed to couples carrying different mutations and only aimed to the exclusion of the paternal allele in the enriched fetal cells, as most of the times they were contaminated from maternal cells.

In subsequent years, even if the fetal cells enrichment and selection methods have been greatly improved, other NIP diagnosis have been carried out but with fluctuating results. Below are described three significative examples of NIPD realized, with different level of success, by using single or pooled erythroid cells.

In 1996 the group of Y.W.Kan^47^ reported the successful identification of two fetal genotypes by using fetal nucleated erythroid cells selected by MACS, anti-ζ globin immunostaining and then isolated by microscopy and cell scraping. The presence/absence of sickle cell and beta thalassemia mutations of both parents were finally detected by Reverse Dot Blot in PCR amplified samples constituted by pools of fetal dissected cells.

Few years later the group of Di Naro^48^ replicated this results using a slightly different procedure for erythroblast enrichment which was carried out by Percoll and Gastrografin multiple gradient centrifugation. Mutation detection was then obtained by automated sequencing of single cells amplified by PCR. According to authors, even if the risk of allele drop out is higher when amplifying single cells, however the possibility to study several individual, instead of pooled, cells guarantees an accurate diagnosis of the fetal DNA.

More recently the group of Kolialexi^49^ has hardly tried to replicate these results. In this study, NIPD was performed through magnetic cell sorting (MACS) and microdissection of single NRBCs with a laser micromanipulation system. Single-cell genotyping was achieved by nested real-time PCR for genotyping β-globin gene mutations; a multiplexed minifingerprinting was used to confirm the origin of the isolated cells and to exclude their possible contamination. A total of 224 cells were isolated but only half of them were successfully amplified. In the majority (n=80) of these cells minifingerprinting was not informative because of allele dropout or homozigosity. In the rest of the samples, 22 cells resulted to be of fetal origin, 26 maternal while 80 were non informative.

### Analysis of Fetal DNA in Maternal Plasma and Non Invasive Prenatal Diagnosis (NIPD) of β Thalassemia:

The existence of cell-free nucleic acids within the human plasma was firstly reported in 1948 by Mendel and Metais^50^ which described their presence both in normal subjects and in individuals affected by various diseases. Some decades later other studies have confirmed the presence of circulating DNA as well as of RNA in several pathological conditions (pancreatitis, inflammatory diseases, cancer, diabetes, etc).^51^

In 1997 Lo et al discovered for the first time that a fetus may release cell-free fetal DNA (cffDNA) into maternal plasma, thus providing an alternative to fetal cells for noninvasive prenatal diagnosis.^52^

In recent years more information have been acquired about the concentration, the origin and the characteristics of the cell-free fetal DNA and several procedures have been developed in order to use it in prenatal diagnosis.

The cell free DNA is constantly present in peripheral blood of non pregnant women and its concentration increases during pregnancy. The cell-free fetal DNA represent the 3–5% of the DNA present in maternal plasma from which, after delivery, it is rapidly cleared.

Recent studies carried out by microfluidic digital PCR have revealed that cffDNA can be present at even higher concentrations which can reach up to 10–20% of total DNA in maternal plasma.^53^ Nevertheless, because of the high background of maternal DNA, an enrichment step is needed to obtain highly purified fetal DNA samples suitable for non invasive prenatal diagnosis.

Size-fractionation agarose gel electro-phoresis is one of the methods developed for fetal DNA enrichment and consists in the isolation of short-length DNA fragments (< 300 bp of length) which is the medium length of the cffDNA. This method coupled with the peptide-nucleic-acid clamp (PNA) PCR, which selectively suppresses the amplification of maternal alleles, and with the Allele-specific Real-Time PCR for mutation detection, has been used with success by Li et al^54^ to detect mutations of paternal origin in fetuses at risk for β-thalassemia.

More recently^55^ the same group has described a new procedure, still based on size fractionation method, but coupled with MALDI-TOF mass-spectrometry, a medium-throughput platform which detect with high sensitivity the presence of known and unknown point mutations. In this case no suppression of maternal allele was carried out and the molecular diagnosis was addressed to the exclusion of the paternal mutated allele. The analysis by MALDI-TOF preceded by size fractionation has given improved results, in comparison to the absence of enrichment, in the detection of the codon 39 β-thalassemia paternal allele.

Nevertheless, for eventual future diagnostic application the protocol needs to be validated in larger sample, even if the high cost of the instrumentation required makes this platform difficult to apply in routine diagnostics and the size fractionation is considered an enrichment method more susceptible to maternal contamination.

The use of peptide-nucleic-acid clamping to suppress the amplification of normal maternal alleles was firstly described by Cremonesi in 2004.^56^ Peptide nucleic acid are artificially synthesized polymers similar to nucleic acids and able to hybridize DNA sequences. The PNA/DNA hybrids are more stable than equivalent DNA/DNA hybrids but less stable in the presence of single-pair mismatches. In that paper their ability to clamp wild type β-globin sequences was proved in artificial mixture of DNA samples enriched with increased amounts of wild type alleles, by using a microchip platform to detect the β-thalassemia mutations.

Four years later^56^ the efficacy of PNA was evaluated with success in 41 non invasive prenatal diagnosis of β-thalassemia and in combination with three different techniques (microelectronic chip, pyrosequencing and direct sequencing) to detect fetal DNA mutations in maternal plasma.

Despite its successfully application, this strategy, as the other above described technologies, is still restricted to couples which carry different mutated alleles and aimed to the detection of mutated paternal alleles.

Another method recently described for NIPD of β-thalassemia is called APEX namely Arrayed Primer Extension. This is a mutation detection system which is based on the combined use of the microchip technology and the single nucleotide base extension method. This system has been recently described by the group of Papasavva^57^ and used to characterize the presence of the paternal β-thalassemia mutations and associated β-globin gene SNPs, in cffDNA isolated from maternal plasma. The possibility to study the polymorphisms associated to the mutated alleles represent a feature of great value since it would give the possibility to extend NPID to couples which carry the same mutated allele. Prerequisite for its application is to find informative SNPs associated with parental mutations which can help to discriminate the paternal mutated allele and to characterize the haplotypes inherited from the fetus. The authors of the paper described the correct application of this methodology in the NIPD of six out of seven couples at risk for β-thalassemia, carried out in the Cypriot population.

## Future Perspective:

As previously reported, one of the major problem which still limits the application of the described protocols in clinical practice is the impossibility to obtain highly purified fetal, cellular as well as cffDNA, samples which could allow the detection of parental alleles, even when they are identical. Few clinical applications of NIPD are actually restricted to the detection of the Y chromosome, for fetal sex determination, or the Rhesus D gene, in Rhesus D negative women, or, in general, of genetic loci which are absent in the maternal genome.

In recent years a great improvement has been obtained in the field of the technologies which can explore the presence of sequence variations even in single molecules of DNA. The concept of “Digital PCR” was firstly in introduced in 1992 by Sykes^58^ who described a method to determine the number of starting DNA template by doing Poisson statistical analysis of PCR results obtained in limiting dilutions. The more recent development of the emulsion PCR (emPCR) have further enhanced the possibility to study single molecules of DNA by using small volume of reactions, water-oil emulsions and microfluidic as well as high-throughput platforms (for a review of both methods and application to NIPD please see Zimmermann et al.^59^

Recent applications of these technologies in the field of NIPD, and in particular in the diagnosis of aneuploidies and monogenic disorders, have shown that these methodologies may find useful application in the near future, even if several drawbacks need to solved and wider validation studied should be carried out before transferring their use in routine diagnostics.
